# Identification and Functional Analysis of the Vision-Specific BBS3 (ARL6) Long Isoform

**DOI:** 10.1371/journal.pgen.1000884

**Published:** 2010-03-19

**Authors:** Pamela R. Pretorius, Lisa M. Baye, Darryl Y. Nishimura, Charles C. Searby, Kevin Bugge, Baoli Yang, Robert F. Mullins, Edwin M. Stone, Val C. Sheffield, Diane C. Slusarski

**Affiliations:** 1 Department of Biology, University of Iowa, Iowa City, Iowa, United States of America; 2 Howard Hughes Medical Institute, Chevy Chase, Maryland, United States of America; 3 Department of Pediatrics, University of Iowa, Iowa City, Iowa, United States of America; 4 Department of Obstetrics and Gynecology, University of Iowa, Iowa City, Iowa, United States of America; 5 Department of Ophthalmology and Visual Sciences, University of Iowa, Iowa City, Iowa, United States of America; University of Wisconsin-Madison, United States of America

## Abstract

Bardet-Biedl Syndrome (BBS) is a heterogeneous syndromic form of retinal degeneration. We have identified a novel transcript of a known BBS gene, *BBS3* (*ARL6*), which includes an additional exon. This transcript, BBS3L, is evolutionally conserved and is expressed predominantly in the eye, suggesting a specialized role in vision. Using antisense oligonucleotide knockdown in zebrafish, we previously demonstrated that *bbs3* knockdown results in the cardinal features of BBS in zebrafish, including defects to the ciliated Kupffer's Vesicle and delayed retrograde melanosome transport. Unlike *bbs3*, knockdown of *bbs3L* does not result in Kupffer's Vesicle or melanosome transport defects, rather its knockdown leads to impaired visual function and mislocalization of the photopigment green cone opsin. Moreover, *BBS3L* RNA, but not *BBS3* RNA, is sufficient to rescue both the vision defect as well as green opsin localization in the zebrafish retina. In order to demonstrate a role for *Bbs3L* function in the mammalian eye, we generated a *Bbs3L-null* mouse that presents with disruption of the normal photoreceptor architecture. *Bbs3L-null* mice lack key features of previously published *Bbs-null* mice, including obesity. These data demonstrate that the *BBS3L* transcript is required for proper retinal function and organization.

## Author Summary

Retinitis pigmentosa (RP), a disorder of retinal degeneration resulting in blindness, occurs due to mutations in dozens of different genes encoding proteins with highly diverse functions. To date, there are no effective therapies to delay or arrest retinal degeneration. RP places a large burden on affected families and on society as a whole. We have studied a syndromic form of RP known as Bardet-Biedl Syndrome (BBS), which leads to degeneration of the photoreceptor cells and is associated with non-vision abnormalities including obesity, hypertension, diabetes, and congenital abnormalities of the kidney, heart, and limbs. In this study we utilized two model systems, the zebrafish and mouse, to evaluate the function of a specific form of BBS (BBS3). We have identified a novel protein product of the *BBS3* gene and demonstrated that functional and structural abnormalities of the eye occur when this form of BBS3 is absent. This finding is of significance because it indicates that *BBS3* mutations can lead to non-syndromic blindness, as well as blindness associated with other clinical features. This work also indicates that treatment of BBS3 blindness will require replacement of a specific form of the *BBS3* gene.

## Introduction

Visual impairment and blindness have far reaching implications for society. Hundreds of individually rare, but collectively common Mendelian disorders can cause blindness. One of these disorders is a heterogeneous syndromic form of retinal degeneration, Bardet-Biedl Syndrome (BBS, OMIM 209900). This pleiotropic disorder is characterized by retinal degeneration, obesity, polydactyly, renal abnormalities, hypogenitalism and cognitive impairment [1]–[4]. Additionally, BBS is associated with an increased incidence of hypertension, diabetes mellitus and heart defects [1],[2],[5]. Although there is variability in the ocular phenotype between individuals, BBS patients typically present with early and progressive photoreceptor degeneration, leading to both central and peripheral vision loss by the third decade of life [1], [6]–[13].

To date, 14 genes (*BBS1*-*14*) have been implicated in BBS [14]–[28]. Analysis of mouse models of BBS (*Bbs1^M390R/M390R^*, *Bbs2^−/−^*, *Bbs4^−/−^* and *Bbs6^−/−^*) reveals that these mice have major components of the human phenotype including retinal degeneration, obesity, renal cysts and neurological deficits [29]–[32]. Multiple lines of evidence suggest that BBS phenotypes involve cilia dysfunction in a range of tissues, including the retina. The vertebrate retina contains photoreceptors, highly polarized cells with a modified cilium (connecting cilium) that joins the photosensitive outer segment (OS) to the protein synthesizing inner segment (IS). The connecting cilium transports cellular components from the IS to the OS that are necessary for the structure and function of the OS [33],[34]. Intraflagellar transport (IFT) proteins are important in this intraphotoreceptor transport process as they play a key role in both assembly and maintenance of photoreceptor cells [35]–[37]. Loss of IFT genes in vertebrates leads to abnormal OS development, retinal degeneration and mislocalization of photopigments [36],[38],[39].

Retina phenotypes observed in *Bbs-null* mice are similar to those seen with loss of IFT genes, indicating that BBS proteins play a role in transporting proteins through the connecting cilium into the OS of the photoreceptor. For instance, characterization of the retinal phenotype in the mouse model has shown that photoreceptor death is preceded by mislocalization of rhodopsin [29]–[32],[40]. Recent work with two independently generated *Bbs4-null* mice indicates that Bbs4 proteins play an important role in establishing both correct structure as well as proper transport of phototransduction proteins [40],[41]. In the zebrafish model system, individual knockdown of *bbs* genes results in defects in the ciliated Kupffer's Vesicle (KV) and delayed retrograde transport within the melanosome [26],[42],[43]. Moreover, work in *Caenorhabditis elegans* has shown that *bbs1*, *bbs3*, *bbs5*, *bbs7* and *bbs8* localize to the basal body of ciliated cells and are involved in IFT [23],[44]. Taken together, these data strongly support a role for BBS proteins in intracellular transport and cilia; thus further substantiating a critical role for BBS genes in the specialized connecting cilium of the photoreceptor for cell maintenance and function.

BBS3 is a member of the Ras superfamily of small GTP-binding proteins, which is subdivided into ADP-ribosylation factor (ARF) and ARF-like (ARL) subgroups [45]. The precise function of ARL proteins is unknown, but it has been proposed that they play a role in membrane and/or vesicular trafficking [45]. Work in *C. elegans* indicates that *ARL6* is specifically expressed in ciliated cells and undergoes IFT along the ciliary axoneme [17]. Here we report the identification of a second transcript of *BBS3*, *BBS3L*, and determine that the BBS3L protein product plays an important role in eye structure and function. BBS3L is evolutionally conserved and is unique among BBS gene products as it is expressed predominantly in the eye, suggesting a specialized role in vision. We have established both mouse and zebrafish models to study the function of *BBS3L*, and determined that BBS3L is specifically required for retinal organization and function.

## Results

### Identification of a second BBS3 transcript in human, mouse, and zebrafish

Expressed sequence tag (EST) data for human *BBS3* was compared to the known coding region of the gene. Although most of the ESTs were virtually identical to the *BBS3* reference sequence, a few were found to contain 13 extra base pairs. Interestingly, all ESTs that contained this alternative sequence originated from retina or whole eye libraries, suggesting that this second longer transcript, *BBS3L*, has an expression pattern that is limited to the eye.

**Figure 1:**
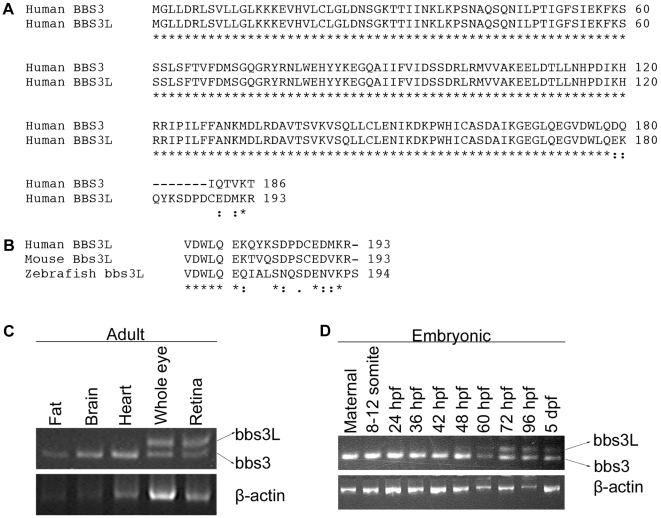
Identification of a second *BBS3* transcript. (A) Alignment of human BBS3 and BBS3L proteins. (B) C-terminal end alignment of human, mouse, and zebrafish BBS3L protein. Asterisks (*) indicate identical amino acids shared in all alignments, while colons (:) and periods (.) represent conserved amino acids. (C) RT–PCR tissue expression profile of zebrafish *bbs3* and *bbs3L* transcripts in wild-type adult zebrafish tissues: fat, brain, heart, whole eye and retina. β-actin was used as a positive control. *bbs3* is expressed in all adult tissues examined, while *bbs3L* expression is limited to the eye. (D) RT–PCR developmental expression profile of *bbs3* and *bbs3L* at the following stages: maternal, 8–12 somites, 24, 36, 42, 48, 60, 72, 96 hpf, and 5 dpf. *bbs3* is expressed throughout development, while the long form is only present by 48 hpf, correlating with photoreceptor development.

*BBS3L* results from differential splicing that leads to the inclusion of a 13 base pair exon and a shift in the open reading frame generating different C-terminal regions (Figure 1A). The striking conservation of the C-terminal region of the long isoform in human, mouse and zebrafish strongly suggests that bbs3L has functional relevance (Figure 1B). To determine if the *bbs3* and *bbs3L* transcripts have similar tissue-specific expression in other species, RT-PCR of zebrafish and mouse tissues was performed. Zebrafish *bbs3* is expressed in all adult tissues examined, while *bbs3L* expression is limited to the eye (Figure 1C). Similar tissue expression patterns for *Bbs3* and *Bbs3L* were seen in the mouse with the addition of low levels of *Bbs3L* mRNA expression in the brain (Figure S1). A developmental profile in zebrafish embryos reveals that while *bbs3* is expressed throughout development, *bbs3L* is not expressed until 48 hours post fertilization (hpf). This is a time when retinal neuroepithelial cells are exiting the cell cycle and differentiating into photoreceptor cells, the light sensing cells of the retina [46] (Figure 1D).

### Knockdown of bbs3 results in characteristic BBS phenotypes

**Figure 2:**
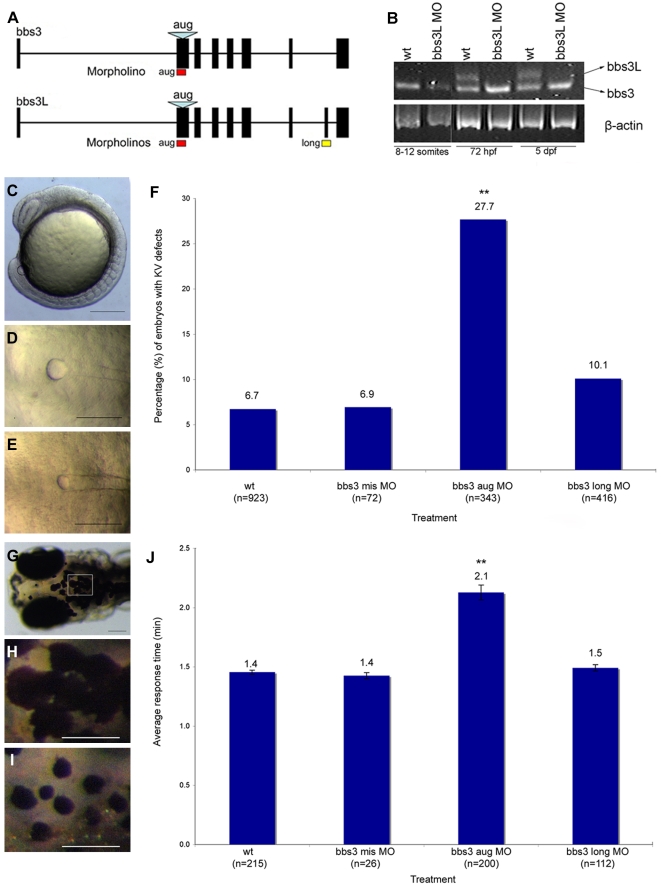
*bbs3* gene structure and cardinal features of BBS knockdown in zebrafish. (A) Schematic depicting the *bbs3* gene structure and antisense oligonucleotide strategy used to target either both transcripts (bbs3 aug MO) or to target only *bbs3L* (bbs3 long MO) in zebrafish embryos. The *bbs3* aug MO targets the start site of the gene and thus hits both transcripts, while the *bbs3L* MO is a splice-blocking morpholino that only targets the long form. (B) RT-PCR from staged *bbs3L* morphant embryos at 8–12 somites, 72 hpf, and 5 dpf. The *bbs3L* transcript is absent through 5 dpf injected embryos indicating successful knockdown. Note that the *bbs3* transcript is unperturbed in *bbs3L* morphants. (C–E) Images of live zebrafish embryos at 8–10 somite stage. Scale bar 200 µm. (C) Side view of an embryo highlighting the location of the Kupffer's Vesicle (circle), the ciliated structure located in the tailbud. (D) Dorsal view of a normal sized KV from a wild-type embryo. (E) *bbs3* aug MO–injected embryos with a reduced KV. (F) The percentage of embryos with KV defects (reduced or absent) in uninjected, control MO, bbs3 aug MO and bbs3 long MO injected embryos. The sample size (n) is noted on the x-axis. **Fisher's Exact test, p<0.001. (G–I) Epinephrine-induced melanosome transport of wild-type 6-day old larvae. Scale bar 100 µm. (G) Melanosome transport is observed in cells on the head of the embryos. Boxed region is magnified for (H,I). (H) Wild-type larvae prior to epinephrine treatment and (I) the endpoint at 1.4 minutes after epinephrine treatment. (J) Epinephrine-induced retrograde transport times. The sample size (n) is noted on the x-axis. **ANOVA with Tukey, p<0.01.

To determine the functional role of bbs3L in development and to distinguish the individual roles of the two bbs3 protein products, we utilized antisense oligonucleotide mediated gene knockdown (morpholinos, MO) in zebrafish. Two independent MOs were utilized: one targeting the splice junction specific to the long transcript (bbs3 long MO) and the other a previously described MO targeting both transcripts (bbs3 aug MO) through blocking of the translational start site [43] (Figure 2A). RT-PCR was used to determine the knockdown efficiency of the bbs3 long MO on staged embryos and demonstrated knockdown of the long transcript through at least 5 days post fertilization (dpf) (Figure 2B).

Knockdown of *bbs* function in zebrafish generates two prototypical defects: reduction of the size of the Kupffer's vesicle (KV) as well as retrograde transport defects [26],[42],[43]. As previously demonstrated, alterations in the formation of the ciliated KV was the earliest observable phenotype resulting from knockdown of both bbs3 transcripts by the bbs3 aug MO [43]. At the 8–10 somite stage (12–14 hpf) in wild-type and control injected embryos the KV has formed in the posterior tailbud. The KV diameter is approximately 50 µm and is larger than the width of the notochord (Figure 2C and 2D). Injection of the bbs3 aug MO resulted in a reduction of KV size to a width less than that of the notochord (Figure 2E). Knockdown of both bbs3 transcripts by the aug MO results in a statistically significant increase in embryos with KV defects (Fisher's exact test, p<0.001) (Figure 2F). Of note, injection of the bbs3 long MO does not lead to KV defects (Figure 2F).

The second prototypical phenotype observed in *bbs* MO-injected embryos (morphants) is delayed trafficking of melanosomes. Zebrafish are able to adapt to their surroundings through intracellular trafficking of melanosomes within melanophores in response to light and hormonal stimuli [47]–[50]. To test the rate of this movement, 5-day old zebrafish were dark adapted, to maximally disperse the melanosomes (Figure 2G and 2H) and then treated with epinephrine to chemically stimulate the retrograde transport of melanosomes [42],[43],[51] (Figure 2I). Wild-type and control injected embryos show rapid movement of melanosomes to a perinuclear location averaging 1.4 minutes, whereas bbs3 aug MO injected embryos demonstrated a statistically significant delay averaging 2.1 minutes (ANOVA with Tukey, p<0.01) (Figure 2J). In contrast, the rate of melanosome movement in bbs3 long knockdown embryos is statistically the same as control embryos, averaging 1.5 minutes (Figure 2J).

To test for MO-specificity as well as differential function of the two *bbs3* transcripts, human RNAs of both *BBS3* and *BBS3L* were used. Embryos co-injected with a combination of MO and RNA were evaluated for suppression of MO induced KV and melanosome transport defects. Co-injection of *BBS3* RNA with the aug MO did not rescue the KV defect but was sufficient to suppress the melanosome transport delay; however, co-injection of the aug MO with BBS3L RNA was not able to suppress either MO-induced defect (Table S1). Myc-tagged *BBS3* and *BBS3L* RNA injection and Western blot analysis confirmed expression of the protein out to 5 dpf (data not shown). Taken together, our data demonstrates that *bbs3L* plays a role independent from KV formation and melanosome transport and that human *BBS3* can partially compensate for the loss of zebrafish *bbs3*.

### *bbs3* long knockdown causes a vision defect in zebrafish

**Figure 3:**
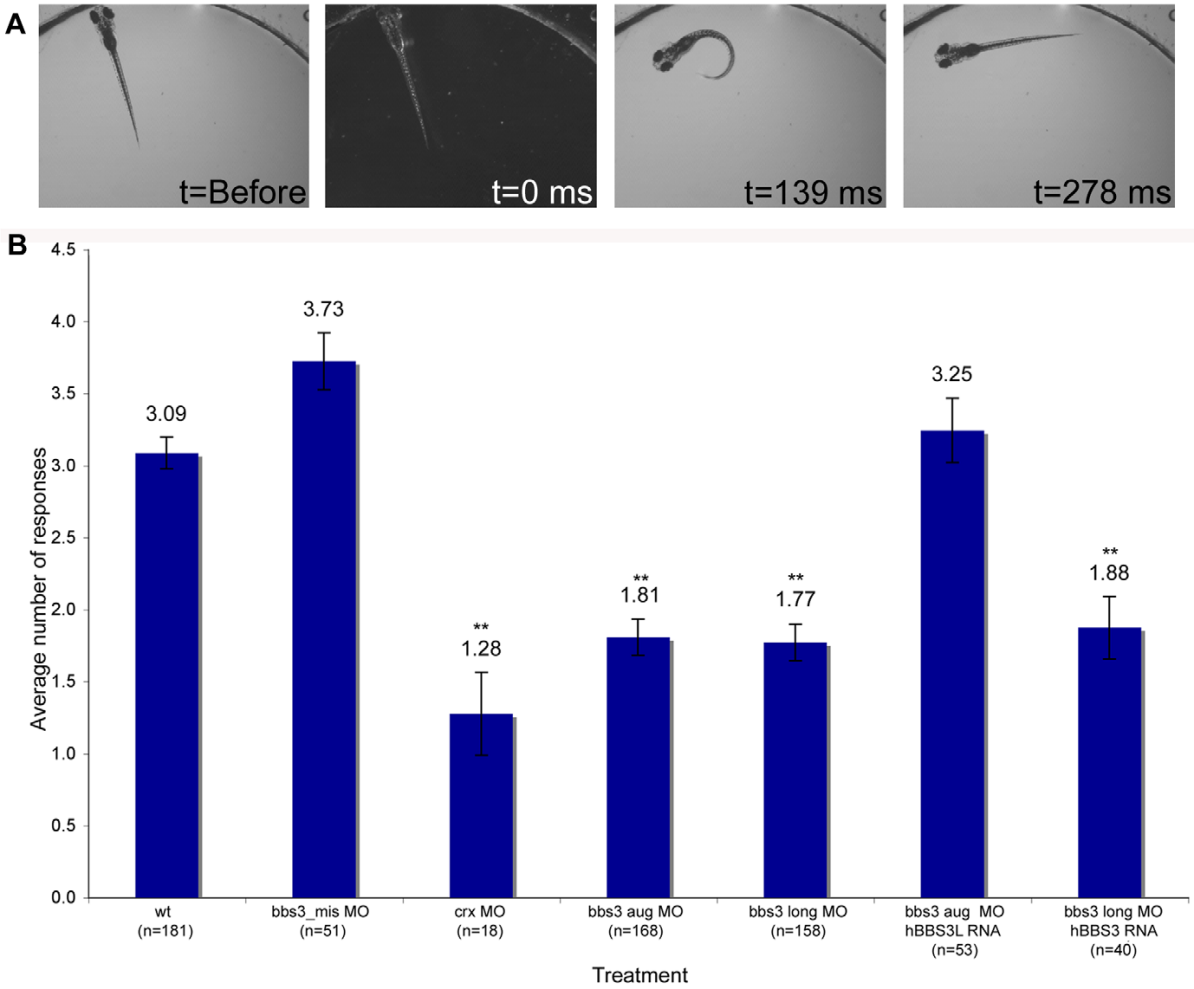
Vision startle response in zebrafish. (A) Vision function was assayed in 5-day old embryos by testing embryos sensitivity to short blocks in light at 30 second intervals for 5 trials (adapted from Easter and Nicola 1996). Selected images from a time-lapse collection before and immediately after a one second block in light. The typical response, a distinct C-bend, is scored as a positive response as shown in time point 139 ms. ms, milliseconds. (B) Quantification of the vision startle response for each treatment. Cone-rod homeobox (*crx*) gene knockdown was used as a control for vision impairment. *bbs3* morphants lacking either both transcripts or only the long transcript showed a statistically significant reduction in the number of responses, indicating visual impairment. Rescue experiments using wild-type human *BBS3L* or *BBS3* RNA co-injected with the bbs3 morpholinos demonstrated that h*BBS3L* RNA is sufficient to rescue the vision defect associated with knockdown, while h*BBS3* is not sufficient to rescue the vision defect. The sample size (n) is noted on the x-axis. **ANOVA with Tukey, p<0.01.

Since BBS patients develop retinitis pigmentosa and the bbs3L transcript is differentially expressed in the eye, we sought to functionally test the role of bbs3 in vision. The zebrafish retina develops rapidly; at 60 hpf the retina is fully laminated and by 3 dpf zebrafish larvae are visually responsive [52]–[54]. Zebrafish elicit a characteristic escape response when exposed to rapid changes in light intensity and this startle response can be used as an assay for vision function [53]. In this assay, the behavior of a 5-day old larvae was monitored in response to short blocks of a bright, stable light source [53] (t = 0, Figure 3A). The typical response, a distinct C-bend and a change in swimming direction, is scored over a series of 5 trials, timed 30 seconds apart (t = 139ms, Figure 3A). Uninjected embryos respond on average 3.09 times (Figure 3B, Table 1 and Video S1). Cone-rod homeobox (*crx*) gene knockdown was used as a control for vision impairment as loss of this gene is known to affect photoreceptor formation in zebrafish [55],[56]. *crx* knockdown embryos respond an average of 1.28 times (ANOVA with Tukey, p<0.01). Knockdown using either the bbs3 aug or bbs3 long MO resulted in a statistically significant (ANOVA with Tukey, p<0.01) reduction in the number of responses (1.81 and 1.77 times respectively) compared to controls, indicating vision impairment (Figure 3B, Table 1 and Video S2). These data support a key role for *bbs3L* in vision function.

**Table 1:** Vision startle assay and percentage of green opsin mislocalization.

Treatment	Vision (# of responses)	n	Mislocalized green opsin (%)	n
wt	3.09	181	10.6	585
bbs3 aug MO	1.81**	168	19.1++	555
bbs3 aug MO + hBBS3 RNA	2.13**	54	20.4++	496
bbs3 aug MO + hBBS3L RNA	3.25	53	12.78	227
bbs3 long MO	1.77**	158	21.0++	905
bbs3 long MO + hBBS3 RNA	1.88**	40	18.8++	330
bbs3 long MO + hBBS3L RNA	2.7	53	10.9	357

** ANOVA and Tukey test, p<0.01 as compared to wt.

++ Fisher's Exact test, p<0.01 as compared to wt.

To functionally test the specific role of both *bbs3* and *bbs3L* in vision, rescue experiments were performed. To investigate whether bbs3 could compensate for loss of *bbs3L*, wild-type human *BBS3* RNA was co-injected with the bbs3 long MO. Although *BBS3* RNA was sufficient to suppress the melanosome transport delays associated with bbs3 aug morphant embryos (Table S1), *BBS3* RNA was insufficient to rescue the vision impairment induced by loss of only *bbs3L* (Figure 3B and Table 1). Conversely, co-injection of *BBS3L* RNA with the bbs3 aug MO was sufficient to rescue the vision defect (ANOVA with Tukey, p<0.01) (Figure 3B and Table 1). Based on these rescue experiments, bbs3L is necessary and sufficient for vision function.

### hBBS3L is sufficient to rescue green opsin mislocalization in bbs3 morphant zebrafish

Previous work has demonstrated that Bbs1 M390R knockin, Bbs2, Bbs4 and Bbs6 mutant mice initially form photoreceptors; however, the photoreceptors subsequently show a mislocalization of rhodopsin, a photopigment protein, to the cell bodies of the outer nuclear layer (ONL) and undergo progressive photoreceptor degeneration [29]–[32],[40]. By gross histology, wild-type, bbs3 aug and bbs3 long morphant zebrafish embryo retinas displayed a fully laminated retina at 5 dpf (data not shown). Ganglion cell outgrowth and optic nerve formation was evaluated using the ath5:GFP [Tg(atoh7:GFP)] transgenic line, a marker of ganglion cell and axon outgrowth [57]. We found that gross retinal ganglion axon trajectories were not perturbed in *bbs3* aug or long morphants (data not shown).

**Figure 4:**
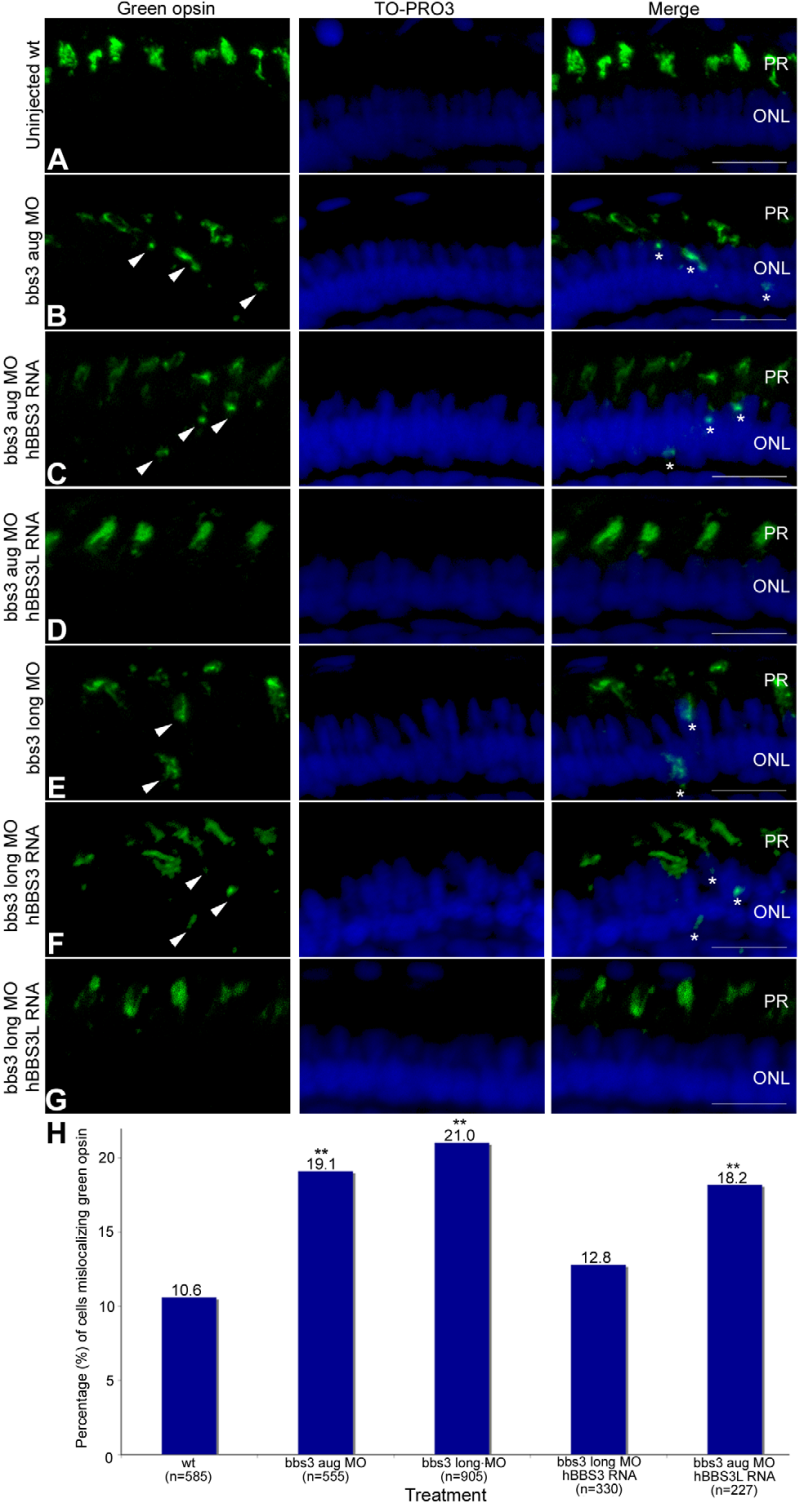
Green opsin mislocalization and rescue in 5-day-old bbs3 morphant zebrafish. Immunofluorescence of green cone opsin (green) on transverse cryosections of 5-day-old embryos (A) uninjected wild-type, (B) bbs3 aug MO, (C) bbs3 aug MO and hBBS3 RNA, (D) bbs3 aug MO and hBBS3L RNA, (E) bbs3 long MO, (F) bbs3 long MO and hBBS3 RNA, and (G) bbs3 long MO and hBBS3L RNA. To-Pro3 was used to counterstain the nuclei (blue). In *bbs3* aug (B) and bbs3 long morphants (E), green opsin was not restricted to the outer segment of the photoreceptors and was detected in the cell bodies of the outer nuclear layer (arrowheads and asterisks). Expression of *hBBS3L* RNA improved green opsin localization in both *bbs3* aug (D) and *bbs3L* (G) morphants. Of note, *hBBS3* RNA failed to rescue green opsin localization in *bbs3* aug (C) and *bbs3* long (F) morphant embryos (arrowheads and asterisks). OS, outer segment; ONL, outer nuclear layer. Scale Bar 10 µm. (H) The percentage of mislocalizing green opsin cells. The sample size (n) is noted on the x-axis and represents the total number of green opsin positive cells counted. **Fisher's exact test, p<0.01.

While the overall architecture of the retina appeared morphologically normal at 5 dpf, we investigated photopigment localization in *bbs3* morphants. Photopigments are known to localize to the outer segment of the zebrafish photoreceptor; therefore, we assessed opsin localization using an antibody specific to green cone opsin [58]. In the wild-type retina, green opsin is found in the outer-segment of the green cone photoreceptor (Figure 4A). In *bbs3* aug and *bbs3* long morphants green opsin expression was not restricted to the outer segments of the photoreceptors; rather, green opsin was also detected in the cell bodies of the outer nuclear layer throughout the entire retina (Figure 4B and 4E).

To determine whether there is a functional difference between *BBS3* and *BBS3L* in its ability to rescue the green opsin localization in the photoreceptors of MO-injected embryos rescue experiments were performed. The first question we addressed was if *BBS3L* RNA was sufficient to rescue green opsin localization in morphant embryos. Expression of wild-type human *BBS3L* RNA led to improved green opsin localization in both *bbs3* aug and *bbs3L* morphant embryos (Figure 4D and 4G). The percentage of cells mislocalizing green opsin was quantified and indeed *BBS3L* RNA was able to statistically rescue the green opsin defect in *bbs3* aug morphants (Fisher's exact test, p<0.01) (Figure 4H and Table 1). We next investigated whether *BBS3* could compensate for loss of *bbs3L* in the zebrafish retina. Co-injection of wild-type human *BBS3* RNA failed to rescue green opsin localization in *bbs3* aug and *bbs3L* morphant embryos (Figure 4C, 4F, and 4H and Table 1). These data are consistent with the vision startle response rescue data and supports the hypothesis that *BBS3L* has an eye specific role. Moreover, these data support a specific role for *bbs3L* in the retina and for localization of proteins within the photoreceptor cell.

### Bbs3 is expressed in ganglion and photoreceptor cells in mouse and human retinas

**Figure 5:**
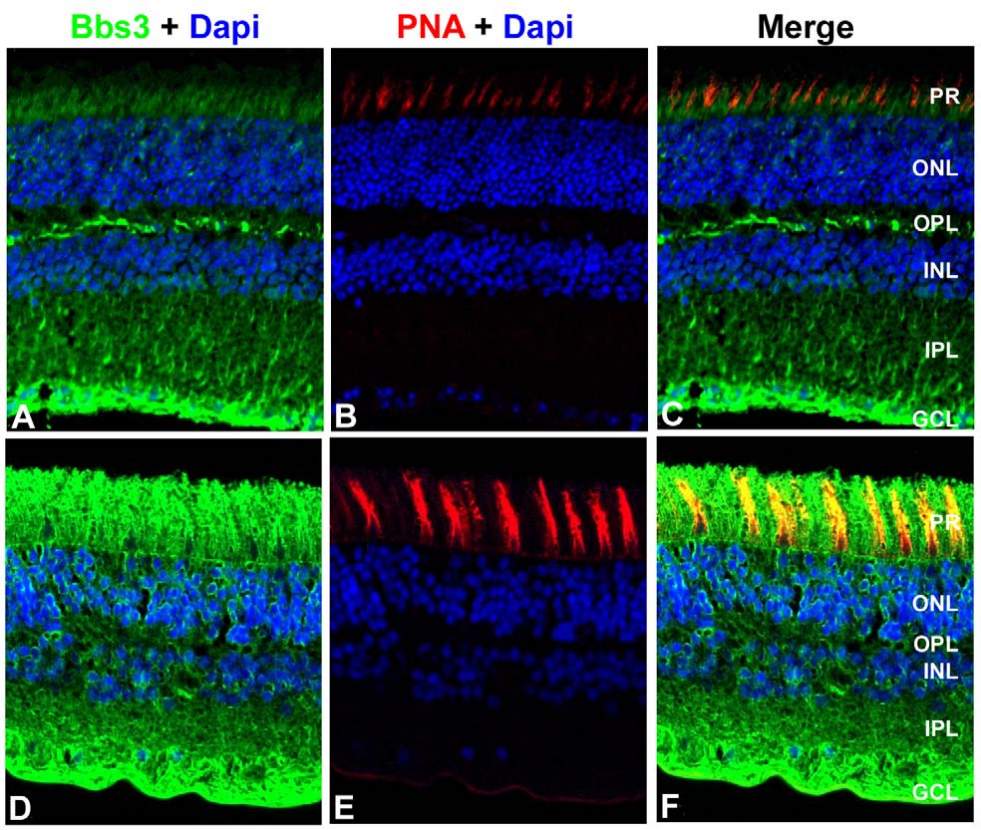
Localization of BBS3 in human and wild-type mouse retinas. Immunohistochemistry triple labeling of cryosections taken from 8-month old wild-type mouse retinas (A–C) and human donor eyes (D–F). Localization of BBS3 (green) using an antibody generated against a central region of the mouse Bbs3 peptide, which recognizes both Bbs3 and Bbs3L (A,D). Peanut agglutinin (PNA, red) was used as a marker for cone outer segments (B,E). Nuclei were counterstained with DAPI (blue). Bbs3 was found robustly in the ganglion cell layer as well as the photoreceptor cell layer of human and mouse retinas. PR, photoreceptor; ONL, outer nuclear layer; OPL, outer plexiform layer; INL, inner nuclear layer; IPL, inner plexiform layer; GCL, ganglion cell layer.

A polyclonal antibody against a central region of the mouse Bbs3 peptide, which is conserved across human and mouse, was generated to recognize both isoforms of Bbs3. Cellular localization of Bbs3 was assessed in donor human and mouse retinal tissue. Immunohistochemistry was performed on transverse cryosections from adult human and adult mouse eyes using the Bbs3 antibody. Staining revealed expression of Bbs3 (green) in the ganglion cell layer and the nerve fiber layer as well as the photoreceptor cells of both mouse (Figure 5A) and human retinal tissue (Figure 5D). Additionally, peanut agglutinin (PNA, red) was used as a marker for cone outer segments in both mouse (Figure 5B) and human retinal sections (Figure 5E). The merge represents the co-localization of Bbs3 (green) and PNA (red) in the photoreceptor cells of both mouse (Figure 5C) and human (Figure 5F). The specificity of the BBS3 antibody for immunohistochemistry was confirmed through peptide blocking of the antibody on wild-type mouse retina (Figure S2).

### *Bbs3L^−/−^* mice display structural abnormalities

**Figure 6:**
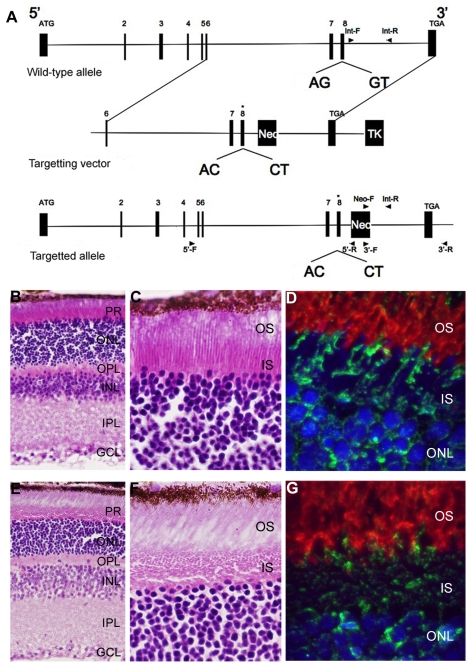
Generation and initial characterization of a *Bbs3L* mutant mice. (A) Schematic for the targeted alteration of the splice donor and acceptor sites of exon 8 (asterisk) found in the Bbs3L transcript. Homologous recombination leads to the inclusion of these altered sites and the loss of Bbs3L. Hematoxylin/eosin staining of cryosections from 8-month old *Bbs3L^+/+^* (B,C) and *Bbs3L^−/−^* (E,F) mouse retinas. Disruption of the normal photoreceptor architecture was observed in *Bbs3L^−/−^* mice. Immunohistochemistry analysis of cryosections from (D) wild-type and (G) targeted mutants retinas using the Bbs3 antibody (green) and rhodopsin (red), a marker for rod photoreceptor outer segments. To-Pro3 was used as a counterstain for nuclei. PR, photoreceptor; ONL, outer nuclear layer; OPL, outer plexiform layer; INL, inner nuclear layer; IPL, inner plexiform layer; GCL, ganglion cell layer.

To characterize the effects of loss of *Bbs3L* on mammalian photoreceptors a targeted knockin of the long form of Bbs3 was carried out by altering the splice donor and acceptor sites flanking exon 8, leading to the exclusion of exon 8 upon homologous recombination (Figure 6A). This approach leads to the preservation of *Bbs3* expression in the *Bbs3L-null* mice. RT-PCR confirmed the generation and transmission of the *Bbs3L* allele in +/− and *−/−* mice (Figure S3). Unlike previously generated BBS knockout mice, which are obese by 7 months of age, Bbs3L*^−/−^* mice do not become obese (data not shown) [29]–[32]. This supports the idea that *Bbs3L* function is restricted to the retina and is consistent with the zebrafish knockdown studies.

Gross histological examination of 8-month-old wild-type and homozygous (Bbs3L*^−/−^*) mutant mice revealed that while all cell layers were present (Figure 6B and 6E), the inner segments of the photoreceptors were disrupted in a majority of the mutant mice as compared to wild-type (Figure 6C and 6F). In wild-type mice, the inner segment layer is arranged in a parallel array; while in the Bbs3L-null mice the parallel arrangement of the IS was eccentric with individual inner segments randomly oriented. Additionally, immunohistochemistry with the Bbs3 antibody (green), which recognizes the endogenous Bbs3 protein that is still present, and rhodopsin (red) in Bbs3L*^−/−^* mice further confirms inner segment disorganization in Bbs3L*^−/−^* mutant mice compared to wild-type (Figure 6D and 6G).

## Discussion

The present study identifies and characterizes the eye-enriched transcript *BBS3L* using both the zebrafish and mouse model systems. While typical BBS genes are ubiquitously expressed and lead to multiple phenotypes in human, mice and zebrafish, *BBS3L* expression is restricted to the eye and serves as a useful tool for understanding the specific pathophysiology of BBS proteins in blinding diseases. By knockdown in zebrafish, we find that *bbs3L* is required for visual function and localization of the photopigment green cone opsin; however, *bbs3L* is dispensable for the cardinal features of BBS in zebrafish, including reduced KV and delayed melanosome transport. Moreover, *BBS3L* RNA, but not *BBS3* RNA, is sufficient to rescue both the vision defect as well as green opsin localization. These data provide strong evidence that *bbs3L* is specifically required for retinal organization and function.

Immunohistochemistry using an antibody that recognizes both Bbs3 and Bbs3L indicates strong expression of the protein in the ganglion cell layer, nerve fiber layer and photoreceptor cells in both human and mouse retinas. By using this antibody on *Bbs3L-null* mouse retinas, we can deduce that *Bbs3* is expressed in both the photoreceptors and ganglion cells. This is consistent with expression data indicating that *Bbs3L* is enriched in the retina.

We have previously demonstrated that knockdown of *bbs* genes in the zebrafish leads to KV defects and melanosome transport delays [26],[42],[43]. As previously reported, knockdown of *bbs3* using the aug morpholino yields both KV and melanosome transport defects; however, knockdown of only *bbs3L* does not affect the KV or melanosome transport. The lack of these cardinal features is not surprising given that *bbs3L* is not expressed at the KV stage and that in adult zebrafish the long transcript is only expressed in the eye. Since *bbs3* and *bbs3L* are identical except for the splicing of the last exon, we cannot technically knockdown *bbs3* alone without affecting *bbs3L*. However, based on rescue data, *bbs3* knockdown alone appears to be responsible for both the KV and melanosome transport defects seen with the aug morpholino. Importantly, bbs3 and bbs3L do not seem to be functionally interchangeable. Forced expression of *BBS3L* RNA, at a time and place where the endogenous transcript is not present, does not rescue the cardinal features of BBS in the zebrafish that result from knockdown of both transcripts. Moreover, over-expression of *BBS3* in the whole embryo cannot restore vision loss resulting from the knockdown of only *bbs3L*. It should be noted that melanosome transport is evaluated after the vision startle assay; therefore, we know that over-expressed BBS3 is functional at the time of the vision assay. Although bbs3 may have some effect on vision that is below the detection level of our assay, we have demonstrated that bbs3L function is both necessary for vision and sufficient to rescue vision loss in the zebrafish.

Similar to the zebrafish results, a *Bbs3L-null* mouse lacks the observed phenotypes of previously published *Bbs-null* mice, such as obesity [29]–[32]. The effect of *Bbs3* in the mouse retina may be more significant as *Bbs3L-null* mice present with only a variable mild disruption of the normal architecture. This indicates that in the mouse retina, *Bbs3* is able to partially compensate for loss of *Bbs3L*. Moreover, the difference in phenotype between zebrafish and mouse could potentially be due to the ratio of cones and rods found in each model system. One hypothesis is that *bbs3L* plays a major functional role in cones, but only a minor role in rods. At the stages examined in the zebrafish, cones are the only functional photoreceptors in the retina, whereas mice have a rod-dominated retina [59],[60]. These attributes are important to consider when looking at the role of BBS in human disease progression, as humans rely on their fovea, a specialized cone-dominant structure in the center of the macula, for visual acuity. Continued characterization of the *Bbs3L-null* mouse may shed more light on this difference between the mouse and zebrafish system, as well as elucidate a more definitive role for *BBS3L* in the retina.

Taken together, these date demonstrate that the *BBS3L* transcript is specifically required for retinal organization and function. While we have identified a second transcript of *BBS3*, a gene known to cause BBS, we would not expect patients with mutations affecting only BBS3L to present with BBS. Based on our findings in both a zebrafish and mouse model of *BBS3L*, patients with mutations in BBS3L alone would present with a non-syndromic retinal disease, characterized by photoreceptor dysfunction and death. Indeed, recent homozygosity mapping of a consanguineous Saudi family has identified a missense mutation in BBS3 that leads to non-syndromic RP [61]. Functional characterization of this mutation in the zebrafish may provide additional clues to the role of BBS3 in the eye. Thus this eye specific transcript, *BBS3L*, will serve as a useful tool for understanding the pathophysiology of other blinding diseases. In addition, our data indicate that expression of *BBS3L*, rather than *BBS3*, would be needed for gene therapy aimed at treatment of blindness in BBS3 patients.

## Materials and Methods

### Ethics statement

All animal work in this study was approved by the by the University Animal Care and Use Committee at the University of Iowa.

### EST

Expressed sequence tag (EST) data for human and mouse *BBS3* was downloaded from NCBI and compared to the known coding region as represented by the NCBI reference sequence (NM_177976.1 and NM_032146.3 for human and NM_019665.3 for mouse).

### *Danio rerio*

#### RT–PCR

RNA was extracted from a pool of 10–20 embryos at the following stages: 8–12 somites, 24, 36, 42, 48, 60, 72, 96 hpf and 5 dpf. Additionally, RNA was extracted from the following adult tissues: fat, brain, heart, whole eye and retina. cDNA was synthesized using oligo dT primers and bbs3 primer pair 1 recognizing both *bbs3* transcripts were used to evaluate expression. β-actin expression served as a control.

Primers:

bbs3 primer pair 1-F: 5′-AAGGACAAACCATGGCATATC-3′

bbs3 primer pair 1-R: 5′-TTACGTTTTCATCGCTCTGAT-3′

β-actin-F: 5′-TCAGCCATGGATGATGAAAT-3′

β-actin-R: 5′-GGTCAGGATCTTCATGAGGT-3′

#### Morpholino injections and knockdown efficiency

Antisense morpholinos (MO) were designed and purchased from Gene Tools.

*bbs3_aug* [43]: AGCTTGTCAAAAAGCCCCATTTGCT

*bbs3_long*: ATTTCAGCTTCAGTACTTACAGTGC

control MO *bbs3_6mis*: AaCTTGTgAAAtAGCgCCATaTGaT

*crx:* GGCTGCTTTATGTAGGACATCATTC

MOs (12 ng) were air-pressure-injected into one- to four-cell staged embryos. Transcript knockdown efficiency was assessed by RT-PCR as described above using bbs3 long splice-blocking morphants at the following stages: 8–12 somites, 72 hpf and 5 dpf. Primers recognizing both *bbs3* (bbs3 primer pair 1) transcripts were used to assess knockdown efficiency.

#### Human BBS3 cloning and RNA synthesis

Wild-type human BBS3 and BBS3L constructs were generated by TA cloning into the Gateway vector system (Invitrogen), and subsequently subcloned into Gateway expression vectors with a C-terminal mCherry or myc tag (generous gift from Chien and Lawson Lab).

Primers:

-hBBS3-F: ATGGGATTGCTAGACAGACTTTC

-hBBS3-R: TGTCTTCACAGTCTGGATCTG

-hBBS3L-R: TCTTTTCATGTCTTCACAGTC

For rescue experiments, MO-resistant C-terminally tagged mCherry RNA was synthesized using the mMessage mMachine transcription kit (Ambion). *hBBS3* or *hBBS3L* RNA (8 pg) was co-injected with the appropriate MO into one- to four-cell staged embryos.

#### Analysis of Kupffer's Vesicle

Embryos with KVs smaller than the width of the notochord (less than approximately 50 µm in diameter) were considered reduced, while embryos in which KVs could not be morphologically identified were scored as absent. Live embryos were photographed on a stereoscope with a Zeiss Axiocam camera.

#### Melanosome transport assay

The melanosome transport assay was performed as previously described [26],[42],[43]. Dark-adapted 5-day-post fertilization larvae were treated with epinephrine (50 mg/ml, Sigma, E4375) added to egg water [62] for a final concentration of 500 µg/ml. Melanosome retraction time was monitored under the microscope. Live embryos were photographed on a stereoscope with a Zeiss Axiocam camera.

#### Vision startle response assay

A visually evoked startle response behavioral assay was modified from a previously described assay [53]. Prior to experimentation, 5-day-old zebrafish larvae were light adapted for 1 hour. A visual stimulus was applied by performing rapid changes (approximately 1 second) in white light intensity through abruptly opening and closing the shutter located between the light source and the animal. An abrupt movement of the zebrafish within one second of visual stimuli application was scored as a positive response. After performing five visual stimuli trials spaced at 30 seconds apart, the mechanical stimulus response was evaluated by probing embryos with the tip of a blunt needle. Embryos that failed to respond to the mechanical stimulation, although rare, were not included in the analysis.

#### Immunohistochemistry

Five day post-fertilized larvae were fixed overnight at 4°C with 4% paraformaldehyde (PFA) prepared in BT buffer (4% sucrose, 0.1M CaCl_2_ in 0.1M PO_4_, pH 7.3). Embryos were rinsed with phosphate-buffer saline (PBS) and infiltrated at 4°C with 15% sucrose, 30% sucrose and overnight in 100% optimal cutting temperature compound (OCT, Sakura). Embryos were cryosectioned at −21°C. Sections were collected at 12 µm and were allowed to dry for 1 hour at 25°C. The tissues were incubated with blocking solution (5% normal donkey serum, 0.1% tween-20, 1% DMSO in PBS) for 2 hours and then incubated overnight at 4°C with mouse-anti-green cone opsin diluted in blocking solution (1∶500, generous gift from the Hyde lab). Following washes with PBDT (PBS, 1% DMSO, 0.1% tween-20) sections were incubated for 1.5 hours at 25°C with goat-anti-mouse Alexa 488 (1∶400, Molecular Probes) diluted in blocking solution. Nuclei were counterstained with To-Pro3 (1∶1000, Molecular Probes) diluted in PBS. Sections were mounted in Vectashield mounting medium (Vector Laboratories) and analyzed using a Leica SP2 laser confocal microscope system with 63× magnification and 3x zoom. Images are representative of maximum projections of multiple focal planes (z-series).

#### Green opsin cell counts

The ratio of mislocalized green opsin cells to total green opsin positive cells was determined from a 12 µm thick central retina image taken of a single eye. Mislocalization of green opsin was defined as the presence of green opsin present in the outer nuclear layer (ONL) of the retina. The number of independent fish retinas counted per group were as follows: wt *n = 9*, bbs3 aug MO *n = 10*, bbs3 long MO *n = 15*, bbs3 aug MO+ hBBS3L RNA *n = 5*, bbs3 long MO+hBBS3 RNA *n = 5*. Scorers were masked to the genotype of the embryos.

### *Mus musculus*

#### Generation of Bbs3L mutant mice

A targeting plasmid was constructed by amplifying the 5′ and 3′ regions of *Bbs3L* using genomic DNA isolated from the 129/SvJ mouse strain. The consensus splice sites were ablated to alter the slice donor (5′- CTGCTGTCACAAAAAACAGTACACTAAGTATCTG-3′) and splice acceptor (5′- CAGATACTTAGTGTACTGTTTTTTGTGACAGCAG-3′) sites flanking exon 8, the exon responsible for the long transcript. Following mutagenesis, these regions were cloned into the targeting vector pOSDUPDEL (a gift from O. Smithies, University of North Carolina, Chapel Hill, NC, USA). The targeting construct was linearized with *Not*I and electroporated into R1 embryonic stem (ES) cells (129 X 1/SvJ3 129S1/Sv). Double selection of ES cells was carried out for the presence of the neomycin gene (Neo) and the absence of the thymidine kinase gene (TK). To identify *Bbs3L*-targeted ES cells, G418-resistnt clones were screened for by PCR. One ES cell line was used to produce chimeras that were bred with C57BL/6J mice to generate Bbs3L heterozygous (*Bbs3L^+/−^*) mice on a mixed background. These mixed background offspring were evaluated for germline transmission by PCR, and the resulting Bbs3L heterozygotes (*Bbs3L^+/−^*) crossed to pure 129/SvEv mice for seven generations to enrich for the pure 129/SvEv background. Heterozygous mice were intercrossed and the progeny genotyped by PCR using primers to identify the presence of the targeted allele. Presence of the wild-type and mutant allele was determined by using a three primer pool: forward primer specific to the wild-type allele (5′-TTGGAGATTTGTCTCCCTCTG-3′), forward primer specific to the mutant allele (5′-GCTACCCGTGATATTGCTGAA-3′) and a reverse primer that recognizes both alleles (5′-AAAAGGGCATAAAAGCACCTC-3′).

#### Histological analysis of *Bbs3L^−/−^* mice

Enucleated eyes were fixed in 4% PFA in PBS (pH 7.4). Following 2–4 hours of fixation, the anterior chamber and lens of the eye was removed and the eyecup allowed to fix further overnight at 4°C in 4% PFA. The eyecups were rinsed with PBS and cryoprotected through a series of 5%:20% sucrose incubations (2∶1, 1∶1, 1∶2) before an overnight incubation at 4°C in 20% sucrose. Eyecups were infiltrated with 2 parts 20% sucrose in 1 part OCT (Sakura) for thirty minutes at room temperature. Sections were collected at 7 µm and were allowed to dry for at least 1 hour at 25°C. Gross morphology of the retinas was evaluated with hematoxylin/eosin staining.

#### Immunohistochemistry of *Bbs3L^−/−^* mice

Immunohistochemistry was performed on cryosections from *Bbs3L^+/+^* and *Bbs3L^−/−^* mouse retinas. The sections were blocked with bovine serum albumin (BSA, 1 mg/ml) in PBS for 15 min and then incubated for 1 hour at room temperature with either rabbit anti-mouse Bbs3 (1∶100), biotinylated peanut agglutinin (1∶100, PNA, Vector Laboratories) or monoclonal mouse rhodopsin (1∶1000, RET-P1, NeoMarker) in PBS. Following washes with PBS, sections were incubated for 30 minutes at room temperature with a species specific secondary antibody: goat-anti-rabbit Alexa 488 (1∶200, Molecular Probes), Texas Red Avidin D (1∶200, Vector Laboratories) or goat-anti-mouse Alexa 546 (1∶200, Molecular Probes). Nuclei were counter stained with either 4′, 6-diamidino-2-phentlindole (DAPI, Molecular Probes) or To-Pro-3 (1∶1000, Molecular Probes). Sections were mounted in Aqua Mount (Lerner Laboratories) and analyzed using either an Olympus BX-41 microscope with a SPOT RT digital camera (Diagnostic Instruments) or a Bio-Rad 1024 confocal microscope system. Images from the confocal are representative of multiple focal planes (z-series).

## Supporting Information

Figure S1 Expression of Bbs3 and Bbs3L in wild-type mouse tissues. RT-PCR run on a silver stained denaturing gel used to initially identify the long transcript of *Bbs3* in mouse tissues.
(0.22 MB TIF)

Figure S2 Bbs3 antibody blocking with peptide. Peptide blocking was used to further confirm the specificity of the Bbs3 antibody on wild-type mouse tissue. PR, photoreceptor; ONL, outer nuclear layer; OPL, outer plexiform layer; INL, inner nuclear layer; IPL, inner plexiform layer; GCL, ganglion cell layer.
(0.67 MB TIF)

Figure S3 Expression of *Bbs3* and *Bbs3L* in *Bbs3L*-targeted mice. RT-PCR analysis of *Bbs3* and *Bbs3L* expression in the whole eye from heterozygous (+/−), homozygous (−/−) and wild-type (+/+) mice.
(0.32 MB TIF)

Table S1 Percentage of abnormal KV and melanosome transport times.
(0.09 MB TIF)

Video S1 A response wild-type zebrafish embryo. Real-time imaging of 5-day-old zebrafish embryos during the vision startle response assay. The dark frames correspond to the lights being turned off. Wild-type embryo demonstrating an immediate response to the change in light intensity.
(6.07 MB AVI)

Video S2 A non-responsive bbs3L morphant embryo. Real-time imaging of 5-day-old zebrafish embryos during the vision startle response assay. The dark frames correspond to the lights being turned off. bbs3 long knockdown embryo that does not respond to the change in light intensity, but does respond to mechanical stimulation with a blunt needle.
(7.31 MB AVI)
