# Supplementary figures and images for: Identification and Functional Analysis of the Vision-Specific BBS3 (ARL6) Long Isoform
Source: PLoS Genet. 2010 Mar 19;6(3):e1000884. doi: 10.1371/journal.pgen.1000884 (PMC2841623; doi:10.1371/journal.pgen.1000884)

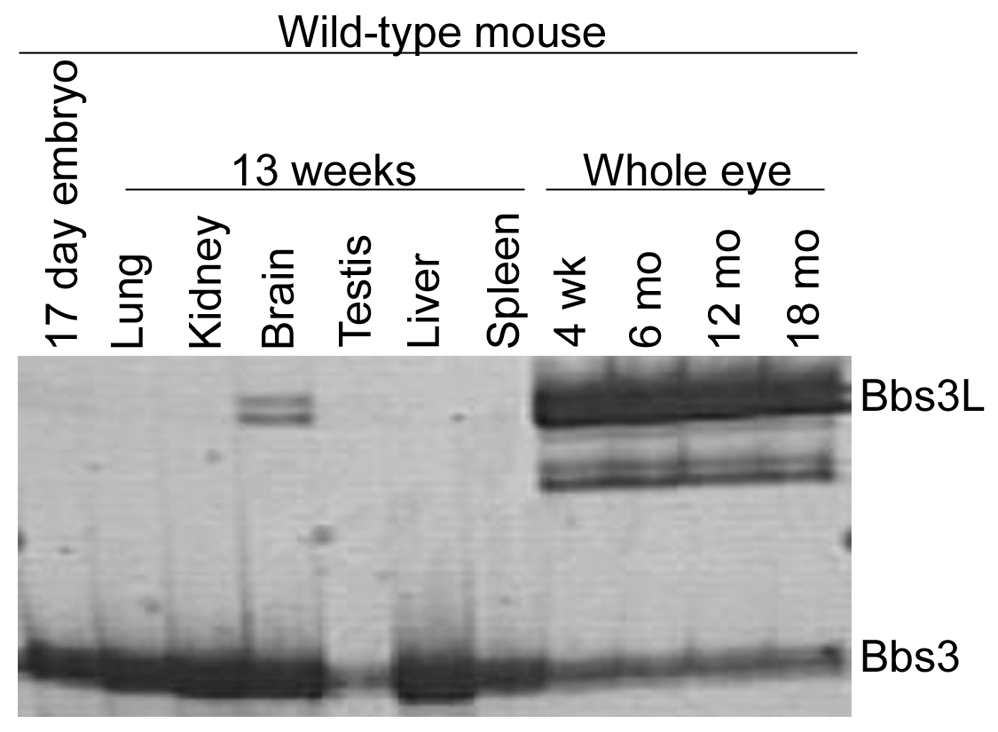

Supplement: Figure S1 — Expression of Bbs3 and Bbs3L in wild-type mouse tissues. RT-PCR run on a silver stained denaturing gel used to initially identify the long transcript of Bbs3 in mouse tissues. (0.22 MB TIF) [file pgen.1000884.s001.tif]

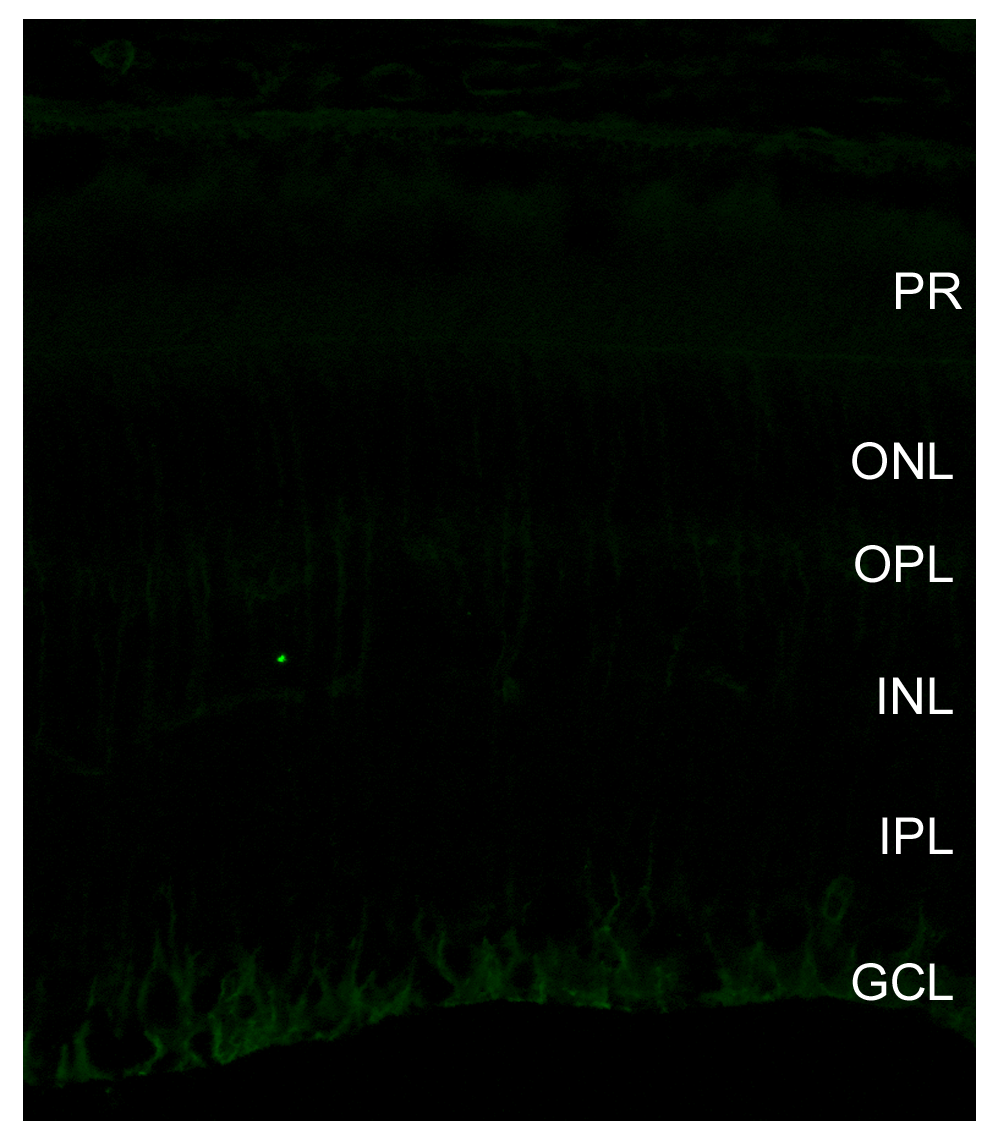

Supplement: Figure S2 — Bbs3 antibody blocking with peptide. Peptide blocking was used to further confirm the specificity of the Bbs3 antibody on wild-type mouse tissue. PR, photoreceptor; ONL, outer nuclear layer; OPL, outer plexiform layer; INL, inner nuclear layer; IPL, inner plexiform layer; GCL, ganglion cell layer. (0.67 MB TIF) [file pgen.1000884.s002.tif]

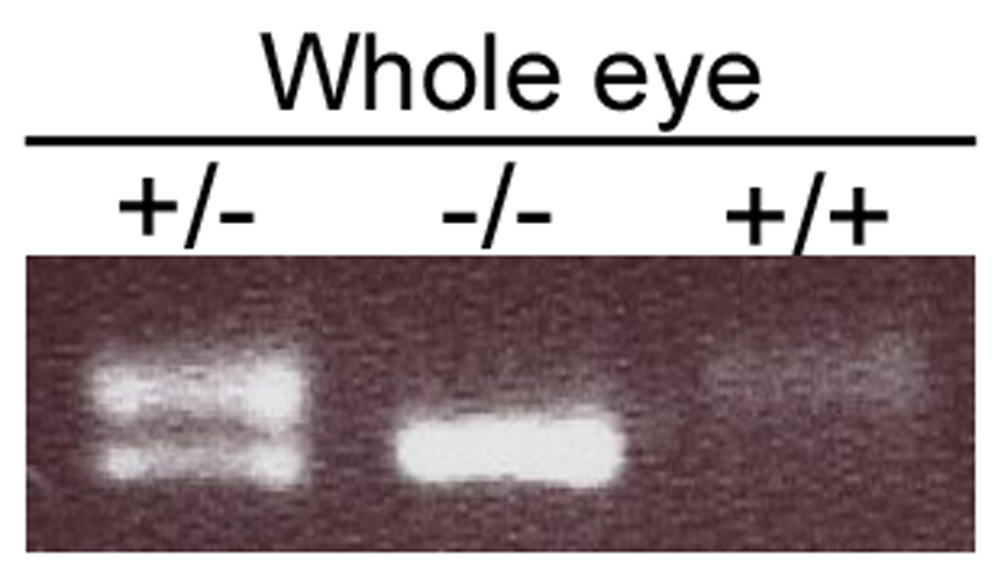

Supplement: Figure S3 — Expression of Bbs3 and Bbs3L in Bbs3L-targeted mice. RT-PCR analysis of Bbs3 and Bbs3L expression in the whole eye from heterozygous (+/−), homozygous (−/−) and wild-type (+/+) mice. (0.32 MB TIF) [file pgen.1000884.s003.tif]

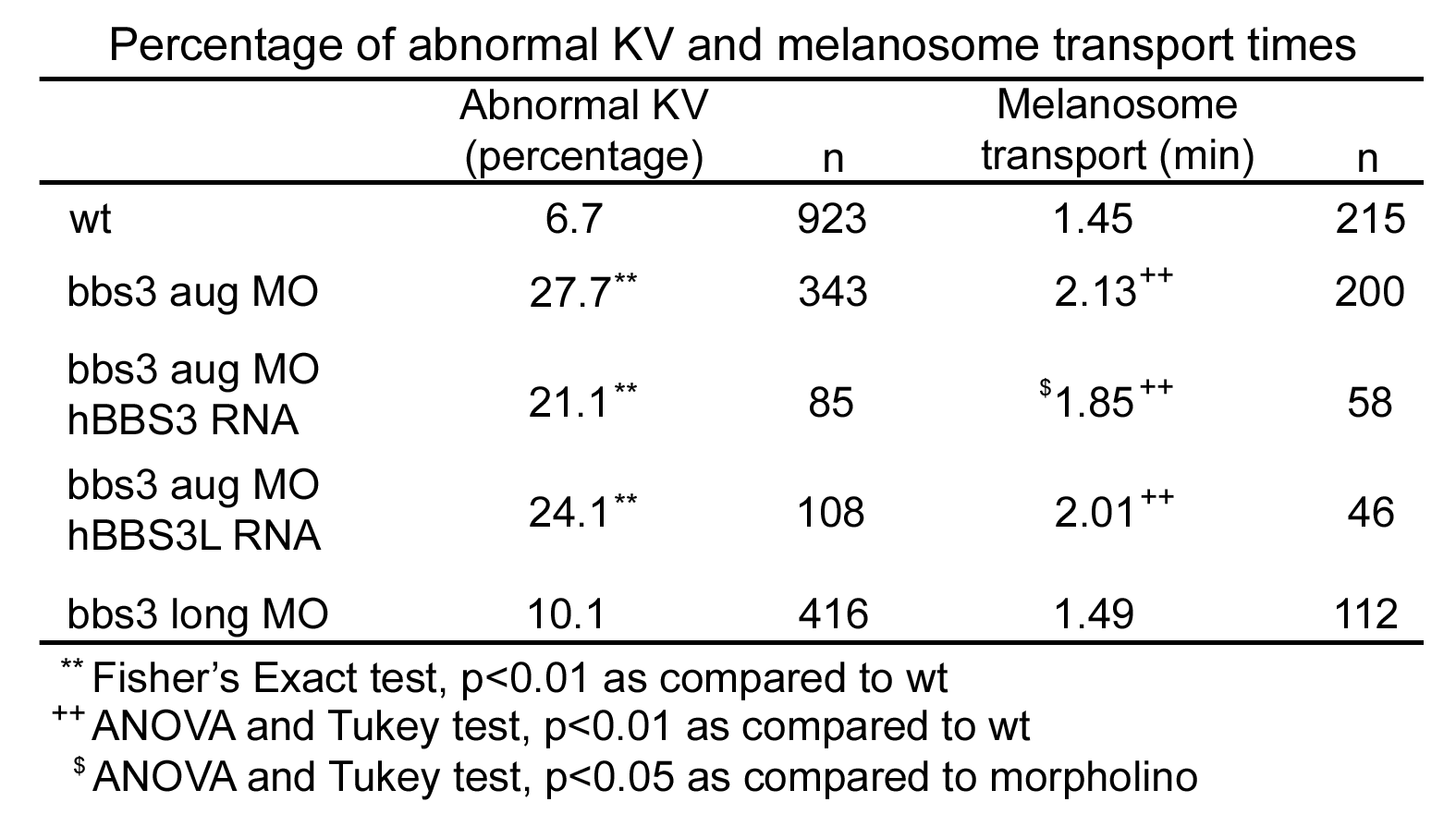

Supplement: Table S1 — Percentage of abnormal KV and melanosome transport times. (0.09 MB TIF) [file pgen.1000884.s004.tif]
